# Effects of the Interaction Between Oxidative Balance Score and Polygenic Risk Scores on Incidence of Metabolic Syndrome in Middle-Aged Korean Adults

**DOI:** 10.3390/antiox13121556

**Published:** 2024-12-18

**Authors:** Minyeong Kim, Dayeon Shin

**Affiliations:** Department of Food and Nutrition, Inha University, 100 Inha-ro, Michuhol-gu, Incheon 22212, Republic of Korea; kmy000923@naver.com

**Keywords:** metabolic syndrome, oxidative balance score, polygenic risk score

## Abstract

Oxidative stress is implicated in insulin resistance, obesity, and metabolic syndromes (MetSs). However, the interplay between oxidative stress and genetic predisposition during the development of MetS remains unclear. In this study, we aimed to investigate the effects of the interaction between oxidative balance score (OBS) and polygenic risk score (PRS) on the incidence of MetS in middle-aged Korean adults. We analyzed data from 25,879 participants aged ≥40 years from the Health Examinees Cohort of the Korean Genome and Epidemiology Study. The OBS was calculated using 11 antioxidant and five pro-oxidant factors. A genome-wide association study and clumping analysis identified 16 independent single-nucleotide polymorphisms associated with MetS that were used to calculate individual PRSs. Multivariable Cox proportional hazard models adjusted for confounding variables were used to assess the impact of OBS and PRS on the incidence of MetS. During a mean follow-up period of 4.3 years, we recorded 3153 cases of MetS. In both men and women, the group with the lowest OBS and a high PRS had a 1.50-fold (hazard ratio [HR] 1.50, 95% confidence interval [CI] 1.07–2.11) and 1.89-fold (HR 1.89, 95% CI 1.40–2.56) higher incidence, respectively, of MetS compared to those with the highest OBS and a low PRS. Among women with a high PRS, the HRs decreased significantly across OBS quintiles 1 through 5 (*p* for trend = 0.009). These findings suggest that managing the oxidative balance may be particularly crucial for individuals with a high genetic risk for MetS.

## 1. Introduction

Metabolic syndrome (MetS) is defined as a combination of several key risk factors for cardiovascular diseases, such as hypertension, increased blood sugar levels, low high-density lipoprotein (HDL) cholesterol levels, and high triglyceride levels [1]. The fundamental risk factors of MetS are a high-calorie and low-fiber diet, a Western diet, and reduced physical activity [2,3,4]. MetS can result in conditions such as type 2 diabetes mellitus, atherosclerosis, stroke, and other disorders, making it a significant public health concern given its increasing global prevalence [2]. In Korea, the prevalence of MetS has increased noticeably from 27.1% in 2001 to 33.2% in 2020. Although the prevalence of MetS has decreased slightly in women (28.2–26.2%), it has increased significantly in men (25.8–40.0%) [5]. Similar increasing trends have been reported in other Asia–Pacific countries [6]. In 2021, the prevalence of MetS in Chinese adults was 32.97% based on the International Diabetes Federation criteria and 29.75% based on the National Cholesterol Education Program Expert Panel (NCEP)–Adult Treatment Panel III (ATP III) criteria [7]. In a study of Vietnamese adult employees who underwent health checkups from 2020 to 2022, the prevalence of MetS, according to the NCEP-ATP III–Asia criteria, was highest (49.1%) in those aged 60 years or older [8]. Additionally, according to a recent study that examined data from the National Health and Nutrition Examination Survey (NHANES) from 1999 to 2020, 42.6% of American adults have MetS [9]. In 2021, the global burden of chronic diseases was the highest for hypertension at 226 million disability-adjusted life years (DALYs), followed by obesity at 129 million DALYs and type 2 diabetes at 75 million DALYs [10]. Therefore, the need to prevent and manage MetS is becoming increasingly strong based on these statistics. To reduce the economic burden related to global public health, it is essential to implement preventive strategies, including a healthy diet, regular physical activity, and early diagnoses and management.

MetS is also closely associated with oxidative stress. Oxidative stress occurs when reactive oxygen species (ROS) levels exceed the capacity of the biological defense systems, thereby contributing significantly to the mechanisms underlying MetS [11]. Humans possess an antioxidant defense system designed to neutralize ROS; however, oxidative damage can occur when internal and external stressors overwhelm this system [12,13]. Increased ROS levels can be caused by various environmental stressors including high-fat, high-carbohydrate meals [14,15], chronic inflammation [16], pollution [17], and cigarette smoke [18,19]. Patients with MetS typically experience increased oxidative stress and impaired antioxidant defenses [20]. Excess ROS production can exacerbate pathological conditions, including a range of chronic conditions such as obesity and insulin resistance [11]. Additionally, high-fat intake may interfere with normal metabolic processes and potentially increase oxidative stress. After a high-fat meal, oxidized cholesterol derivatives known as oxysterols are produced through lipid peroxidation, leading to the generation of cytokines that induce inflammation. Increased cytokine production activates inflammatory cells and increases oxidative stress [21]. Antioxidant nutrients primarily include compounds such as vitamins A, C, and E; zinc; and manganese, which are essential for preventing cell damage caused by free radicals and nitrogen, thereby reducing the likelihood of developing chronic conditions [22]. A study by Wei et al., which was conducted with 2069 participants, indicated a negative correlation between vitamin C consumption and MetS. Using the quartile with the lowest vitamin C consumption as a reference, a tendency toward reduced MetS occurrence was observed in the highest-consumption quartiles [23]. Among Korean participants aged 30–60 years, males in the group with higher intakes of retinol, carotenoids, and vitamin E showed a significantly lower occurrence of MetS than those in the lower-intake group. Among women, a lower incidence of MetS was observed in groups consuming medium and high levels of retinol, whereas those with a high intake of vitamin A showed a reduced likelihood of developing abdominal obesity [24]. In Korean women, when total vitamin A and C consumption doubled, the prevalence of MetS decreased by 5.8% and 6.7%, respectively [25]. Thus, oxidative stress is associated with MetS and its components, whereas the consumption of antioxidant nutrients is associated with a low risk of developing MetS.

MetS can be influenced not only by dietary changes, lifestyle habits, and environmental factors but also by genetic factors. Recent genomic studies have provided important information for understanding the pathogenesis of MetS and developing personalized prevention and treatment strategies. These studies suggest the possibility of early detection of a genetic predisposition to MetS and prevention of disease onset through appropriate lifestyle interventions. Several genetic variations linked to MetS have been identified through genome-wide association studies (GWASs), contributing to our understanding of how these variations affect metabolic pathways [26]. Recent advances in genetic research have also led to the development of polygenic risk scores (PRSs), which combine the effects of multiple genetic variants to predict an individual’s risk of developing MetS. *FTO*, *APOA5*, *APOC3*, *IL6*, *TCF7L2*, and *CETP* contain the most-studied single-nucleotide polymorphisms (SNPs) associated with MetS [27]. The *IRS1*, associated with insulin resistance, and the *MC4R*, associated with abdominal obesity, are strongly associated with MetS [28]. People with the AA genotype of *Sirt1* have a 2.41 times increased occurrence of MetS than those with the GG genotype [29]. For the *FTO* gene variant, rs9939609, which is strongly linked to obesity, Korean women with the A allele have an incidence of obesity 1.28 times higher than those with the TT genotype [30]. Thus, MetS is associated with genetic factors, and understanding its complex pathogenic mechanisms requires an integrated approach that considers dietary habits, lifestyle, and environmental and genetic factors.

MetS is associated with both oxidative stress and PRSs. However, the interplay between oxidative stress and genetic predisposition to the development of MetS remains unclear. Furthermore, studies employing prospective cohort designs to clarify the association between oxidative stress-related MetS and PRS in Korean adults are lacking. Therefore, we hypothesized that the interaction between the oxidative balance score (OBS) and PRS affects the occurrence of MetS in middle-aged Korean individuals.

## 2. Materials and Methods

### 2.1. Study Design and Population

We used data from the Health Examinees (HEXA) cohort of the Korean Genome and Epidemiology Study (KoGES). The HEXA cohort of the KoGES was used to study the impact of environmental and genetic factors on chronic conditions in the Korean population. The participants of KoGES-HEXA aged 40 and above. A baseline survey of the HEXA cohort was conducted at 38 medical facilities across eight different regions in Korea from 2004 to 2013, and follow-up data were collected from 2012 to 2016. Follow-up data were collected regularly via mail and telephone calls to participants. A detailed explanation of this method was provided in previous studies [31,32].

**Figure 1:**
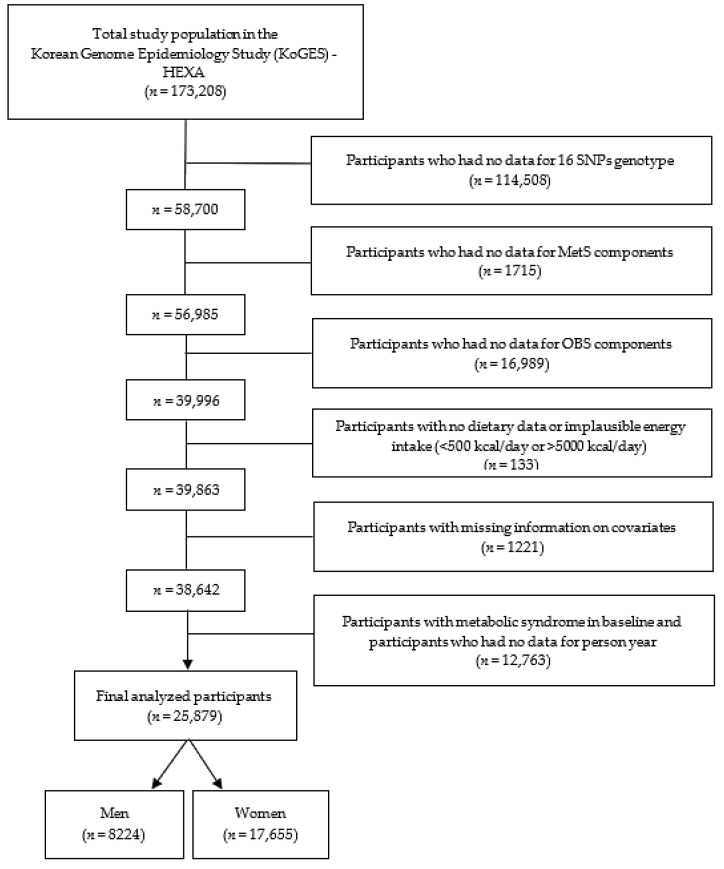
A flow diagram of the study design and participant enrolment, including the number of participants and the exclusion criteria.

Figure 1 presents a flowchart of the study sample enrolment. A total of 173,208 participants in the KoGES-HEXA cohort were excluded because they did not have genotyping data for the 16 SNPs (*n* = 114,508); they did not have data on MetS components (*n* = 1715); they did not have data on the OBS components (*n* = 16,989); they had inaccurate energy intake data (<500 kcal/day or >5000 kcal/day; *n* = 133); they had incomplete information regarding covariates (*n* = 1221); they were diagnosed with MetS at baseline; or they did not have person-years data (*n* = 12,763). Ultimately, 25,879 participants (8224 men and 17,655 women) were included in this study.

### 2.2. Data Collection and Covariates

Height and weight were recorded with the participants barefoot and wearing lightweight clothing. Body mass index (BMI) was calculated by dividing the individual’s weight by the square of height (kg/m^2^). The classification of BMI was conducted following the guidelines of the Korean Society for the Study of Obesity (KSSO, 2022) [33], which defines obesity as a BMI ≥ 25 kg/m^2^. Waist circumference was measured horizontally at the midpoint between the lowest rib and iliac crest. Blood pressure was measured using a mercury sphygmomanometer (Baumanometer; WA Baum, Copiague, NY, USA) after 10 min of rest in the sitting position. Blood samples were drawn in the morning after a 12 h fasting period. Enzymatic assays were used to measure the concentrations of fasting plasma glucose, triglycerides, and HDL cholesterol using a chemistry analyzer (Hitachi 7600; Hitachi, Tokyo, Japan, until August 2002, and ADVIA 1650; Siemens, Tarrytown, NY, USA, from September 2002).

Analyses were performed after adjusting for variables, such as age, educational level, income level, menopausal status, marital status, total energy intake, level of regular exercise, and BMI. Participants were classified according to educational level as elementary school or below, middle school, high school, or university or above. Participants were classified by income level as low income (<1 million KRW/month), middle income (1–3 million KRW/month), or high income (≥3 million KRW/month). Menopause status was classified as pre-menopausal, if answering “yes” to the question “Have you had menstruation in the last 3 months?”, and as postmenopausal if answering “no” to this question. Marital status was classified as single, married, or other. Regular exercise was categorized as active in response to the question “Do you exercise routinely to the point where your body sweats?” and as inactive if answering “no” to this question.

### 2.3. Definition of MetS

Anthropometric measurements corresponding to the MetS-associated components were obtained using data from the KoGES-HEXA cohort. Participants with three or more specified risk factors were classified as meeting the standards for MetS according to the guidelines established by the U.S. NCEP-ATP III (2001) [34] and KSSO (2022) [33]. Abdominal obesity was defined as a waist circumference ≥90 cm for men and ≥85 cm for women, based on the KSSO guidelines. Elevated blood pressure was defined as a systolic blood pressure ≥130 mmHg, a diastolic blood pressure ≥85 mmHg, a diagnosis of hypertension, the use of antihypertensive drugs, or receiving continuous treatment for hypertension. Hypertriglyceridemia was defined as a triglyceride level ≥150 mg/dL. Elevated fasting blood glucose level was defined as ≥100 mg/dL or a diagnosis of diabetes, use of diabetes medication, or continuous treatment for diabetes. Low HDL cholesterol levels were defined as <40 mg/dL in men and <50 mg/dL in women.

### 2.4. Dietary Assessment

Dietary intake was assessed using a 106-item semiquantitative food frequency questionnaire specifically developed and validated for the Korean population. Participants reported the average frequency of food consumption and the amount of food consumed in the previous year. The daily intake of total energy (kcal/day), dietary fiber (g/day), carotene (μg/day), riboflavin (mg/day), niacin (mg/day), vitamin B6 (mg/day), total folate (mcg/day), vitamin C (mg/day), vitamin E (alpha-tocopherol equivalents [35]) (mg/day), calcium (mg/day), zinc (mg/day), total fat (g/day), and iron (mg/day) for each participant was calculated using the nutrient database CAN-pro (version 5.0) developed by the Korean Nutrition Society.

### 2.5. OBS Assessment

OBS was calculated based on dietary and lifestyle factors, as indicated in previous studies [36], by summing the levels of 11 antioxidant factors and 5 pro-oxidant factors. Antioxidant factors included dietary intake of fiber, carotene, riboflavin, niacin, vitamin B6, total folate, vitamin C, vitamin E (ATE), calcium, and zinc along with physical activity. The daily intakes of dietary fiber, carotene, riboflavin, niacin, vitamin B6, total folic acid, vitamin C, vitamin E (ATE), calcium, and zinc were categorized by sex into the following tertiles: high intake, 2 points; medium intake, 1 point; and low intake, 0 points. Physical activity was assigned 2 points for very active (5 times per week to daily), 1 point for active (1–4 times per week), and 0 points for inactive.

Pro-oxidant factors included total fat intake, iron intake, drinking status, smoking status, and BMI. The daily intake of total fat and iron was categorized into tertiles by sex, with 2 points assigned for low intake, 1 point for medium intake, and 0 points for high intake, based on a scale. Drinking status was scored as follows: 2, non-drinkers; 1, former drinkers; and 0, current drinkers. Participants were classified as non-smokers (2 points) if they had never smoked or smoked fewer than 100 cigarettes in their lifetime. Those who had smoked >100 cigarettes but had quit smoking were categorized as former smokers (1 point), whereas those who had smoked >100 cigarettes and were still smoking were classified as current smokers (0 points). The participant’s BMI was assigned 2 points for a low BMI (men ≤ 23.23 kg/m^2^, women ≤ 22.26 kg/m^2^), 1 point for a moderate BMI (men 23.24–25.47 kg/m^2^, women 22.27–24.67 kg/m^2^), and 0 points for a high BMI (men > 25.47 kg/m^2^, women > 24.67 kg/m^2^). Therefore, the sum of the OBS scores was 0–32 points.

Individual OBSs of the participants were classified into quintile groups according to sex. For men, the mean OBS was 8.00 (range, 3.00–9.00) for quintile 1, 11.00 (range, 10.00–13.00) for quintile 2, 15.00 (range, 14.00–17.00) for quintile 3, 20.00 (range, 18.00–21.00) for quintile 4, and 23.00 (range, 22.00–30.00) for quintile 5. For women, quintile 1 was 9.00 (range, 4.00–10.00), quintile 2 was 12.00 (range, 11.00–14.00), quintile 3 was 17.00 (range, 15.00–19.00), quintile 4 was 22.00 (range, 20.00–23.00), and quintile 5 was 25.00 (range, 24.00–30.00).

Higher OBS scores were assigned when there were higher levels of antioxidant factors or lower levels of pro-oxidant factors, whereas lower OBS scores were assigned when there were lower levels of antioxidant factors or higher levels of pro-oxidant factors. Therefore, low OBS may reflect a state of relatively high oxidative stress, potentially contributing to increased inflammation. As OBS is an indicator of the degree of oxidative stress suppression, a low score may indicate a disruption in the antioxidant balance [37].

### 2.6. Genotyping

The GWAS was conducted using the Korean Biobank Array (Korean Chip, Seoul, Korea) to identify SNP genotypes that were significantly associated with MetS in the KoGES-HEXA cohort [38]. The significant threshold for SNP selection was defined as a *p*-value < 5 × 10^–8^ [39]. In the GWAS, the total number of significant SNPs on chromosomes 1 to 22 was 775. A clumping analysis was performed using the PLINK 1.9 software (https://www.cog-genomics.org/plink/1.9, accessed on 28 June 2024) according to the Korea Disease Control and Prevention Agency Guidelines for Polygenic Risk Scores. Finally, we selected 16 independent SNPs (linkage disequilibrium [40] r^2^ < 0.1) from 775 significant SNPs [41]. The properties of the 16 genetic variants satisfying these criteria, namely, rs112405902, rs28869508, rs10503669, rs10830963, rs1787701, rs79408961, rs567346980, rs11216125, rs651821, rs11216140, rs6589574, rs111884008, rs56156922, rs9926440, rs2303790, and rs429358, are detailed in Table 1.

**Figure 2:**
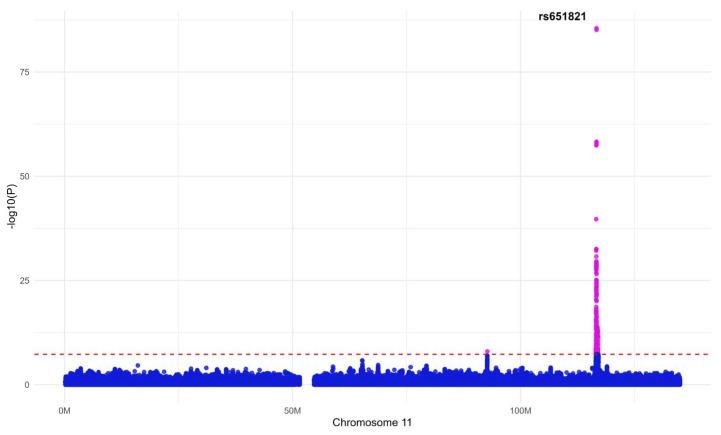
A Manhattan plot of major single nucleotide polymorphisms (SNPs) associated with metabolic syndrome (MetS). This plot represents the association between the SNP (rs651821) with the highest *p*-value among the 16 SNPs selected through genome-wide association studies (GWAS) and the clustering analysis and MetS. The cutoff point is *p* = 5 × 10^−8^, and statistically significant and insignificant SNPs are expressed in two colors based on this cutoff point.

Figure 2 shows the association between MetS and the SNP with the highest *p*-value among the 16 SNPs selected through GWAS and the clumping analysis. This SNP belongs to *APOA5*, a gene closely related to triglycerides [42,43].

### 2.7. Construction of Weighted PRS

**Figure 3:**
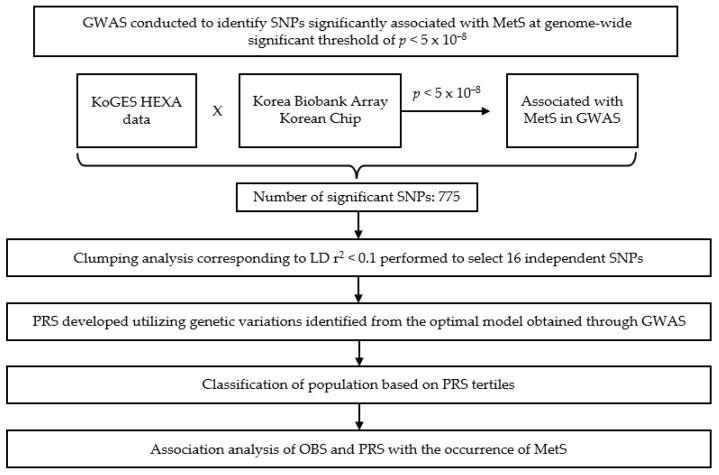
A flowchart of genome-wide association study (GWAS)-based variant identification, polygenic risk score (PRS) construction, and interaction analysis with oxidative balance score (OBS) in metabolic syndrome (MetS). GWAS, genome-wide association study; SNP, single nucleotide polymorphism; PRS, polygenic risk score; MetS, metabolic syndrome; OBS, oxidative balance score; KoGES, Korea Genome and Epidemiology Study; HEXA, Health Examinees; LD, linkage disequilibrium.

A PRS was created by combining the risk alleles of the 16 SNPs identified through the association analysis with MetS. Individual weighted PRSs were calculated by multiplying the number of risk alleles an individual possessed, *risk allele_i_*, by the corresponding weighted odds ratio (OR) coefficient, *weighted OR_i_*, and then summing these values for all 16 SNPs according to Equation (1) [44,45]. Figure 3 shows 16 statistically significant independent SNPs associated with MetS on a chromosome-by-chromosome basis.

(1) $$weighted\;PRS=\sum_{i=1}^{16}\left({risk\;allele}_{i}\times {weighted\;OR}_{i}\right)$$

The PRSs were classified into low, middle, and high tertiles according to sex. The mean PRSs for men in the low, middle, and high tertiles were 4.28 (range, 0.00–5.32), 6.21 (range, 5.32–7.24), and 8.51 (range, 7.24–17.77), respectively, while those for women were 4.24 (range, 0.82–5.37), 6.34 (range, 5.37–7.30), and 8.57 (range, 7.30–17.69), respectively.

### 2.8. Statistical Analysis

The participants’ demographic characteristics were compared by classifying them based on their MetS status and PRS. Continuous variables were expressed as the mean ± standard deviation using Student’s *t*-test and a general linear model. Categorical variables are expressed as frequencies (%) using the chi-square test. GWAS was performed using PLINK version 1.9 software (https://www.cog-genomics.org/plink/1.9, accessed on 28 June 2024).

The OBSs of men and women were divided into five groups (quintiles) to analyze their relationship with MetS, and the PRSs were divided into three groups (tertiles) to analyze their relationship with MetS. In addition, a statistical trend analysis was conducted to determine whether OBS and PRS had a linear relationship with MetS occurrence.

Multivariable Cox proportional hazard models were used to ascertain the hazard ratios (HRs) and 95% confidence intervals (CIs) for the relationships between OBS and MetS, PRS and MetS, and OBS-based PRS and MetS. Age, educational level, income level, menopausal status, marital status, total energy intake, level of regular exercise, and BMI were adjusted for in the analyses. All statistical analyses, except the genetic analyses, were performed using Statistical Analysis System (SAS) version 9.4 (SAS Institute, Cary, NC, USA), and a *p*-value less than 0.05 was considered statistically significant.

## 3. Results

### 3.1. Characteristics of the Study Participants Based on the Presence of MetS

Table 2 shows the demographic characteristics of the study participants based on the presence or absence of MetS. The average follow-up period was 4.3 years, and 3153 cases of MetS were recorded. The participants who completed the follow-up survey and were free of MetS at baseline were divided into two groups: those who developed MetS (*n* = 3153) and those who did not (*n* = 22,726). A significant difference in PRS between men and women (*p* < 0.05) was noted. The MetS group had a high proportion of participants with high PRS, whereas the proportion of participants with low PRS was high in the group without MetS (*p* < 0.05). Men in the MetS group were younger than those in the non-MetS group, and women in the MetS group were older than those in the non-MetS group (*p* < 0.05). The MetS group exhibited significantly higher levels of risk factors such as BMI, waist circumference, systolic and diastolic blood pressure, triglyceride levels, and fasting blood sugar levels, but lower HDL cholesterol levels than those in the group without MetS for both male and female participants (all *p* < 0.05). Significant differences in the educational level were observed between the groups but only among women. A higher percentage of women with MetS had lower levels of education, specifically those who had completed elementary or middle school, than women without MetS (*p* < 0.05). The income levels differed between men and women. The proportion in the lowest-income group was significantly lower than those in the middle- and highest-income groups (*p* < 0.05). Significant differences in smoking status between the groups were observed only in men. In both groups, with and without MetS, the proportion of former smokers was significantly higher than the proportions of non-smokers and current smokers (*p* < 0.05). There were significant differences in alcohol consumption between men and women. Among men, the proportion of current drinkers was significantly higher than that of non-drinkers and former drinkers in both MetS groups. Contrastingly, the proportion of non-drinkers was significantly higher among women than among former and current drinkers. Significant differences in physical activity were observed between the groups but only among men, for whom the MetS group had a higher rate of irregular exercise than the group without MetS (*p* < 0.05). Menopausal status showed observed significant differences between groups. The MetS group had a higher postmenopausal rate than the non-MetS group, and both groups had higher postmenopausal and premenopausal rates (*p* < 0.05).

### 3.2. Characteristics of the Study Participants Based on PRS Tertiles

Table 3 shows the demographic characteristics of the study participants based on the PRS tertiles. Triglyceride levels were significantly higher in both men and women with a high PRS than in those with a low PRS (*p* < 0.05). Fasting blood glucose levels differed significantly only among the women. The high-PRS group had higher fasting blood glucose levels than the low-PRS group (*p* < 0.05). HDL cholesterol levels were lower in both men and women in the high-PRS group than in those in the low-PRS group (*p* < 0.05). Income levels differed significantly only among men, with the proportion of the lowest earners being significantly lower than that of the middle or highest earners in all PRS groups (*p* < 0.05). There were no significant differences between the groups for other variables such as age, BMI, waist circumference, systolic blood pressure, diastolic blood pressure, educational level, smoking status, alcohol intake, physical activity, and menopausal status.

### 3.3. Incidence of MetS According to OBS

Table 4 shows the findings of the multivariable Cox proportional hazards model for the incidence of MetS according to the OBS. Among men and women, the incidence of MetS was low in the highest-OBS-quintile group. In multivariable Cox proportional hazards models adjusted for covariates, men in the highest-OBS-quintile group had a 0.83-fold lower risk of developing MetS than men in the lowest-OBS-quintile group (HR 0.83, 95% CI 0.66–1.04, *p* = 0.11). However, this difference was not statistically significant. In multivariable Cox proportional hazards models adjusting for covariates, women with OBSs in quintiles 2 and 3 had a 0.83-fold lower risk of developing MetS than women with OBSs in quintile 1 (HR: 0.83, 95% CI 0.72–0.96, *p* = 0.014; HR 0.83, 95% CI 0.71–0.96, *p* = 0.011, respectively). Furthermore, women with OBSs in quintiles 4 and 5 exhibited a 0.74-fold decreased risk of developing MetS than women with OBSs in quintile 1 (HR: 0.74, 95% CI: 0.63–0.88, *p* = 0.0005; HR: 0.74, 95% CI: 0.61–0.90, *p* = 0.002, respectively).

### 3.4. Incidence of MetS According to PRS

Table 5 shows the HRs and 95% CIs of the association between PRS and MetS. In men, the incidence of MetS was high in the high-PRS group. In multivariable Cox proportional hazards models adjusting for covariates, men with a high PRS had a 1.27-fold higher incidence of MetS than men with a low PRS (HR 1.27, 95% CI 1.10–1.45, *p* = 0.0007). Among the women, the incidence of MetS was higher in the mid- and high-PRS groups. In multivariable Cox proportional hazards models adjusting for covariates, women with middle and high PRSs had a 1.15-fold and 1.28-fold higher incidence of MetS, respectively, than women with a low PRS (HR: 1.15, 95% CI: 1.03–1.29, *p* = 0.014; HR: 1.28, 95% CI: 1.15–1.43, *p* ≤ 0.0001, respectively).

### 3.5. Incidence of MetS According to OBS and PRS

Table 6 shows the HRs and 95% CIs for the association between OBS and the incidence of MetS according to the PRS. In both men and women, the incidence of MetS varied according to OBS and PRS. Particularly, a high PRS was associated with a high incidence of MetS in the OBS quintile 1 group.

Men with a mid PRS and an OBS in quintiles 3 and 4 had a 1.42-fold and 1.55-fold higher incidence of MetS, respectively, than men with a low PRS and an OBS in quintile 5 (HR: 1.42, 95% CI: 1.02–1.97, *p* = 0.037; HR: 1.55, 95% CI: 1.14–2.11, *p* = 0.005, respectively). Men with a high PRS and an OBS in quintile 1 had a 1.50-fold increase in the incidence of MetS compared to men with a low PRS and an OBS in quintile 5 (HR: 1.50, 95% CI: 1.07–2.11, *p* = 0.019). The same trend was observed in the other groups (quintiles 2 and 3), in which PRS was high and OBS was low. In men with low PRS, the HRs decreased linearly from OBS quintiles 1 to 5 (*p* for trend = 0.033).

Women with a high PRS and OBS in quintile 1 had a 1.89-fold higher incidence of MetS than those with low PRS and OBS in quintile 5. The HRs for MetS tended to decrease linearly as the OBS increased from quintile 1 to quintile 5 (HR: 1.89, 95% CI: 1.40–2.56, *p* for trend = 0.009). Additionally, compared to women with a low PRS and an OBS in quintile 5, those with a middle and high PRS and an OBS in quintile 2 had a 1.58- and 1.67-fold increased incidence of MetS, respectively (HR: 1.58, 95% CI: 1.18–2.13; HR: 1.67, 95% CI: 1.25–2.24, *p* for trend = 0.002). For women with the highest OBS, the group with a high PRS had a linear increase in risk of MetS of 1.51-fold more than the group with a low PRS (HR: 1.51, 95% CI: 1.15–1.98, *p* for trend = 0.002).

## 4. Discussion

We investigated the relationship between individual oxidative stress status and genetic factors associated with MetS in middle-aged Korean adults. PRS was positively correlated with MetS in both men and women, and the incidence of MetS increased as PRS increased within the OBS quintiles. Men and women with high PRS and the lowest OBS had 1.50- and 1.89-fold higher incidences of MetS, respectively, than men and women with low PRS and the highest OBS.

The OBS serves as an indicator of oxidative stress levels, and we combined antioxidant and pro-oxidant factors into a single score, considering the different contributions of each component to the risk of MetS. Antioxidant factors included in the score were the dietary intake of fiber, carotene, riboflavin, niacin, vitamin B6, total folate, vitamin C, vitamin E, calcium, and zinc Along with physical activity, whereas the pro-oxidant factors included total fat intake, iron intake, drinking status, smoking status, and BMI. Dash et al. reported that integrating antioxidant and pro-oxidant factors into a single score can provide a more accurate assessment of oxidative stress than using each antioxidant or pro-oxidant factor individually [35].

Previous studies investigated the association between the OBS and MetS. A study by Lee et al. using KoGES data found that oxidative stress and inflammation were associated with MetS and that increased exposure to antioxidants significantly reduced the incidence of MetS [46]. According to another cross-sectional study, OBS demonstrated a strong positive association with MetS components, such as triglyceride levels, blood glucose levels, waist size, and elevated blood pressure, whereas it was negatively associated with HDL cholesterol levels [47]. Several free-radical species produced in the body perform specific functions. Three ROS, superoxide (O_2_^−^), hydrogen peroxide (H_2_O_2_), and nitric oxide (NO), are essential for normal physiological functions; they also promote aging and induce cellular damage in disease states [48]. Pathological conditions, such as adiposity, insulin resistance, hyperglycemic states, persistent inflammation, and dyslipidemia, may result in ROS overproduction [49,50,51]. In the present study, a significant association between OBS and MetS was observed only in women. As OBS increased from quintile 1 to quintile 5, the incidence of MetS decreased 0.83- and 0.74-fold in men and women, respectively. These sex-based differences may be attributable to various biological and hormonal mechanisms. 

According to Kim et al., healthy-weight postmenopausal women with MetS have higher levels of oxidative stress than metabolically healthy overweight/obese postmenopausal women, and postmenopausal women with MetS have higher levels of inflammatory markers [52,53]. Estrogen plays a significant role in antioxidant defense and regulation by enhancing the expression and activity of antioxidant enzymes, including superoxide dismutase and glutathione peroxidase [54]. Conversely, androgens are believed to contribute to oxidative stress by elevating metabolic rate, potentially leading to increased ROS production through increased oxygen consumption [55]. Lipid metabolism and hormonal influences may contribute to the development of MetS, depending on the differences in metabolic outcomes between men and women. There are significant sex differences in lipid profiles and lipid fractions between men and women, and these differences have important implications for risk of cardiovascular disease. For example, in men, increased free testosterone levels were significantly associated with increased LDL cholesterol levels [56], while higher levels of estrogen in women have been shown to promote the synthesis of HDL cholesterol. Estrogens activate endothelial nitric oxide synthase in vascular endothelial cells, thereby promoting vasodilation, improving blood flow, and protecting against cardiovascular disease [57]. 

Sex hormones have different effects on insulin action depending on sex. In men, an inverse association has been observed between testosterone levels and insulin levels [58]. Androgens are hormones produced primarily in the testes of men, the most important of which is testosterone [59]. Testosterone functions by binding to a specific protein called the androgen receptor (AR). However, even in the presence of sufficient testosterone, if there is a deficiency of AR, testosterone cannot exert its function [60]. In men, experimental studies have shown that AR deficiency worsens obesity and glucose intolerance [61], suggesting that androgens may play a role in modulating insulin sensitivity in men. In contrast, in women, estrogen prevents insulin resistance by regulating metabolic processes that maintain energy balance in insulin-sensitive tissues such as fat cells, liver, and muscle [62]. Additionally, transcription factors estrogen receptor 1 and estrogen receptor 2 have been reported to modulate the expression of the *SLC2A4* gene, altering tissue GLUT4 content and ultimately regulating glycemic control [63]. Genetic variations in these receptors may further modulate the body’s response to sex hormones, leading to differences in susceptibility to obesity, insulin resistance, and lipid abnormalities between sexes. 

We confirmed the effect of dietary habits according to sex on the incidence of MetS. In men, the group with a high intake of retinol, carotenoids, and vitamin E had a lower prevalence of MetS, whereas in women, the group with a high intake of retinol had a lower prevalence of MetS [24]. In addition, women consumed more fruits and vegetables and β-carotene than men, and the protective effect of such intake was also more prominent in women. The average age of the women participants was 38 years [64]. Our study involved middle-aged individuals from Korea, and in the case of postmenopausal women, oxidative stress increased because of a reduction in estrogen levels; however, this suggests that a high OBS may help reduce the risk of MetS. The integration of the findings of previous studies with those of the present study indicates that OBS is a potential predictor of MetS risk.

In the present study, we identified genes most closely associated with MetS, including *APOA5*, *SIK3*, *CETP*, and *APOE*. Among these, the expression level of *APOA5* is the most prominent. *APOA5* is located on chromosome 11q23, close to the *APOA1*/*APOC3*/*APOA4* cluster. *APOA5* has the strongest influence on triglyceride metabolism [65]. Several SNPs in the *APOA5* influence plasma triglyceride levels in various populations. In particular, rs662799 and rs3135506 variants are closely associated with plasma triglyceride levels and hypertension [66,67]. A significant link between MetS and rs651821 in the *APOA5* has been identified. Indeed, in Tunisian women, rs651821 is associated with MetS, consistent with earlier discoveries [68]. A clinical study conducted in an ethnic population in China reported that individuals with the TC or CC genotype of the rs651821 variant had a 1.791 times greater incidence of hypertension than those with the TT genotype [69]. In a Korean population with hypertension, the prevalence of MetS doubled to almost 60%, exhibiting an increase corresponding to that of blood pressure [70]. The pathophysiological mechanisms underlying MetS-related hypertension include insulin resistance, adiposity, sympathetic nervous system hyperactivity, and sodium retention [71]. 

The *SIK3*, found to be associated with MetS in this study, is located on chromosome 11 and encodes a protein that regulates glucose and lipid metabolism and neural development [72,73]. This gene has garnered attention as a potential candidate for the treatment of obesity, metabolic disorders, and neurodegenerative diseases. In our GWAS analysis, we found that the minor allele A of rs6589574 in the *SIK3* was associated with a 1.134-fold increased risk of MetS. We identified *CETP* as a gene associated with MetS. In a Korean cohort, the *CETP* variant rs9926440 showed a strong correlation with chronic conditions such as MetS and dyslipidemia [74]. *CETP* exchanges cholesteryl esters and triglycerides between lipoproteins in the plasma. As a result of this transition, low-density lipoprotein (LDL) and very-low-density lipoprotein (VLDL) particles become enriched in cholesteryl esters and reduced in triglycerides, whereas HDL particles become reduced in cholesteryl esters and enriched in triglycerides, which have been associated with an increased risk of atherosclerosis [40,75]. *CETP* activity influences the size and composition of HDL, thereby altering their functionality. Therefore, high *CETP* activity may decrease HDL levels and cause cholesterol accumulation in LDL and VLDL particles, thereby increasing the risk of atherosclerosis [76]. The *APOE*, which is associated with MetS and its components, particularly cholesterol, is located on chromosome 19q13.32. A biobank study in Taiwan identified the *APOE* ɛ2, ɛ3, and ɛ4 variants as the most influential genetic factors affecting total, LDL, and HDL cholesterol levels [77]. A study conducted on Korean men reported that the *APOE*, along with *APOA5*, was significantly associated with triglyceride levels and the prevalence of MetS [78]. These findings suggest that *APOE* contributes to fluctuations in lipid levels and increases the risk of MetS. Therefore, *APOA5*, *SIK3*, *CETP*, and *APOE* identified to be associated with MetS in this study, may influence MetS and its components.

We found that the impact of PRS on MetS development differed according to the OBS. Men in the high-PRS group with OBS in quintile 2 or 3 had the highest likelihood of developing MetS. Men with low PRS and the lowest OBS had a 1.36-fold higher risk of developing MetS than those with the highest OBS. Contrastingly, women with high PRS and OBS in quintile 1 had a 1.89-fold higher risk of developing MetS. In particular, the risk of developing MetS increased significantly as the OBS decreased in women with a high PRS. Recent studies have demonstrated that the effect of PRS on the risk of insulin resistance is more pronounced in individuals with a low consumption of fruits, vegetables, vitamin C, and flavonoids [79]. This suggests that a low intake of antioxidant nutrients may increase the risk of insulin resistance. In a population-based cohort study, participants in the highest OBS quartile had a lower risk of MetS than those in the lowest quartile. Additionally, GWAS-based pathway analysis has identified VEGF signaling, glutathione metabolism, and Rac-1 pathways as biological pathways underlying the association between MetS and OBS [46]. Additionally, in Iranian adults, the rs1333048 genotype and high lyophilic oxygen radical absorbance capacity intake significantly reduced the likelihood of developing MetS [80]. Thus, previous studies demonstrated a tendency toward alignment. However, because the definitions and components of OBS have not been consistent across studies, the results of studies utilizing OBS may differ. To improve the comparability between studies and derive more reliable results, it is necessary to establish a standardized definition and calculation method for the OBS.

Additionally, our findings suggest that dietary interventions such as increased antioxidant intake may help mitigate the genetic risk of MetS. Resveratrol, a natural polyphenolic compound, restores abnormal insulin, insulin-like growth factor, and blood sugar levels by activating the AMP-active kinase and sirtuin pathways [81]. Similarly, the consumption of blueberries, which are rich in antioxidants, has protective effects against atherosclerosis in ApoE-deficient (ApoE^−/−^) mice by reducing oxidative stress [82]. Increased intake of antioxidant-rich foods such as fruits and vegetables has beneficial effects. These findings highlight the potential of personalized nutritional approaches for individuals at high genetic risk.

In addition to dietary interventions, physical activity, alcohol consumption, smoking, and BMI, which are included in the components of the OBS, may be intervention factors. According to a meta-analysis, exercise tends to increase antioxidant indicators and decrease pro-oxidant indicators, regardless of the intensity, quantity, or type of exercise [83]. Two weeks after the onset of abstinence, the levels of malondialdehyde, a marker of oxidative stress, returned to normal levels [84]. By integrating personalized nutrition, exercise programs, and behavioral strategies, tailored interventions can effectively target individuals with high PRS and low OBS, offering a comprehensive approach to reducing MetS risk. This study highlights the importance of dietary and lifestyle modifications in reducing the risk of MetS, particularly in individuals with high genetic predisposition.

This study has several strengths. First, it was an inaugural study to investigate how the interaction between OBS and PRS affects MetS in Koreans. Second, we investigated the sex differences in the associations between PRS, OBS, and MetS. Third, by adjusting for potential confounding variables such as age and BMI, we were able to identify the distinct effect of the interplay between OBS and PRS on MetS. Despite these advantages, this study has some limitations. First, as this was a prospective cohort study, it was not possible to identify the mechanism of interplay. Second, as this study targeted middle-aged Koreans, the results cannot be generalized to other age or ethnic groups. However, the OBS–PRS model has a broader potential applicability. Future studies should investigate whether this interaction influences MetS development in other ethnic groups or populations with different genetic and environmental profiles. Furthermore, it would be valuable to investigate whether this model can be applied not only to MetS but also to other oxidative stress-related diseases such as cardiovascular diseases and diabetes, thereby further expanding its clinical relevance and generalizability. Third, because many participants were excluded, there may be limitations in generalizing the results to the final sample. Therefore, conducting larger studies or considering studies with different population groups is needed. Fourth, although our study accounted for multiple confounding variables, it is still subject to limitations such as a relatively short follow-up period and potential biases inherent in the data collection process. Thus, unmeasured confounders, such as unreported dietary habits (e.g., caffeine, alcohol, and carbohydrate intake) or environmental stressors (e.g., sleep quality, smoking, and stress), may have influenced the study results [47,85,86]. To address these limitations, future research should aim to collect more precise data and explore strategies for controlling these potential confounding variables. Additionally, the genetic markers analyzed in this study were limited. The PRS utilized was based on 16 independent SNPs identified as significantly associated with MetS in a Korean population. While these markers provide valuable insights, focusing solely on this restricted set may not capture the complex genetic architecture underlying MetS. Hence, adopting a more comprehensive genomic approach is imperative to elucidate the multifaceted genetic contributors to MetS. Finally, in-depth exploration of the biological mechanisms of interaction and sex differences are lacking; future research should address this gap.

In this study, low OBS and high PRS influenced the occurrence of MetS in middle-aged Koreans. In summary, a low antioxidant index and variations in PRS may lead to the development of MetS. This suggests that personalized prevention and treatment strategies for MetS can be developed by considering dietary and environmental factors associated with oxidative stress and PRS.

## 5. Conclusions

In this study, we investigated the effects of the interaction between oxidative stress and genetic predisposition on the development of MetS. In both men and women, the groups with a high PRS showed an increased risk of developing MetS, and a low OBS further increased this risk. Results from a prospective cohort study targeting middle-aged Korean adults revealed that for individuals with a high genetic risk, low levels of OBS amplified the detrimental effects on the likelihood of MetS onset. These results suggested that the interaction between genetic factors and oxidative stress may have a significant synergistic effect on the development of MetS. Managing oxidative stress may be a particularly important preventive measure for individuals with a high genetic risk for MetS. The results of this study may provide an effective strategy for preventing and managing MetS.

## Figures and Tables

**Table 1:** Single-nucleotide polymorphisms (SNPs) associated with metabolic syndrome (MetS).

Chromosome Number	SNP	Gene	Functional Consequence	Position (bp)	Effect Allele	MAF	OR	SE	*p*-Value
11	rs651821	*APOA5*	5′ UTR	116,662,579	C	0.299	1.356	0.015	3.28 × 10^−86^
11	rs11216125	-	-	116,612,274	A	0.196	0.828	0.019	2.12 × 10^−23^
16	rs56156922	-	-	56,987,369	C	0.171	0.823	0.020	1.53 × 10^−22^
8	rs10503669	-	-	19,847,690	A	0.124	0.810	0.023	4.39 × 10^−20^
11	rs6589574	*SIK3*	intron	116,730,638	A	0.358	1.134	0.015	5.73 × 10^−17^
11	rs79408961	-	-	116,588,593	T	0.085	1.228	0.025	2.08 × 10^−16^
11	rs567346980	-	-	116,599,069	C	0.014	1.545	0.056	6.83 × 10^−15^
11	rs111884008	*SIK3*	intron	116,768,743	C	0.149	0.857	0.021	1.49 × 10^−13^
11	rs11216140	-	-	116,672,013	T	0.273	1.124	0.016	4.28 × 10^−13^
11	rs1787701	*LGR5*	-	116,563,992	C	0.280	0.890	0.016	1.30 × 10^−12^
16	rs9926440	*CETP*	intron	57,002,663	C	0.313	1.110	0.015	1.40 × 10^−11^
16	rs2303790	*CETP*	missense	57,017,292	G	0.045	0.790	0.037	2.31 × 10^−10^
19	rs429358	*APOE*	missense	45,411,941	C	0.096	1.156	0.024	1.83 × 10^−09^
6	rs28869508	-	intron	32,343,735	T	0.131	1.131	0.021	4.62 × 10^−09^
6	rs112405902	-	-	31,809,504	A	0.071	1.172	0.027	4.90 × 10^−09^
11	rs10830963	*MTNR1B*	intron	92,708,710	G	0.431	1.087	0.015	1.02 × 10^−08^

MAF, minor allele frequency; OR, odds ratio; SE, standard error; UTR, untranslated region.

**Table 2:** Demographic characteristics of study participants based on the presence of metabolic syndrome (MetS).

Variables	Men (*n* = 8224)		*p*-Value	Women (*n* = 17,655)		*p*-Value
	MetS (*n* = 1250)	Control (*n* = 6974)		MetS (*n* = 1903)	Control (*n* = 15,752)	
PRS (n, %)						
Low PRS	389 (31.1%)	2435 (34.9%)	0.0057	583 (30.6%)	5435 (34.5%)	0.0006
Middle PRS	420 (33.6%)	2370 (34.0%)		630 (33.1%)	5201 (33.0%)	
High PRS	441 (35.3%)	2169 (31.1%)		690 (36.3%)	5116 (32.5%)	
Age (years)	53.8 ± 8.2	55.0 ± 8.6	<0.0001	54.5 ± 7.1	52.1 ± 7.6	<0.0001
BMI (kg/m^2^)	25.2 ± 2.4	23.5 ± 2.3	<0.0001	25.1 ± 2.7	22.8 ± 2.5	<0.0001
Waist circumference (cm)	87.2 ± 6.4	82.7 ± 6.5	<0.0001	81.4 ± 6.9	75.7 ± 7.1	<0.0001
Blood pressure						
Systolic blood pressure (mmHg)	126.4 ± 13.3	122.6 ± 13.2	<0.0001	124.8 ± 13.6	118.1 ± 13.8	<0.0001
Diastolic blood pressure (mmHg)	78.7 ± 8.9	76.4 ± 9.1	<0.0001	76.5 ± 8.8	72.7 ± 9.0	<0.0001
Triglyceride level (mg/dL)	155.9 ± 97.7	116.3 ± 67.2	<0.0001	125.5 ± 62.5	94.8 ± 50.0	<0.0001
Glucose level (mg/dL)	98.0 ± 19.3	94.1 ± 17.3	<0.0001	94.9 ± 17.2	89.4 ± 11.7	<0.0001
HDL cholesterol level (mg/dL)	48.0 ± 10.2	52.7 ± 11.7	<0.0001	51.8 ± 10.7	59.3 ± 12.6	<0.0001
Education (n, %)			0.3583			<0.0001
≤Elementary school	114 (9.1%)	559 (8.0%)		432 (22.7%)	2183 (13.9%)	
Middle school	154 (12.3%)	867 (12.4%)		420 (22.1%)	2633 (16.7%)	
High school	519 (41.5%)	2814 (40.4%)		777 (40.8%)	7291 (46.3%)	
≥College	463 (37.0%)	2734 (39.2%)		274 (14.4%)	3645 (23.1%)	
Income (n, %)			0.0037			<0.0001
Lowest	96 (7.7%)	526 (7.5%)		240 (12.6%)	1378 (8.8%)	
Middle	488 (39.0%)	3071 (44.0%)		882 (46.4%)	6980 (44.3%)	
Highest	666 (53.3%)	3377 (48.4%)		781 (41.0%)	7394 (46.9%)	
Smoking status (n, %)			<0.0001			0.5447
Non-smoker	276 (22.1%)	2063 (29.6%)		1840 (96.7%)	15,295 (97.1%)	
Former smoker	533 (42.6%)	3138 (45.0%)		30 (1.6%)	204 (1.3%)	
Current smoker	441 (35.3%)	1773 (25.4%)		33 (1.7%)	253 (1.6%)	
Alcohol intake (n, %)			<0.0001			0.0407
Non-drinker	207 (16.6%)	1448 (20.8%)		1291 (67.8%)	10,289 (65.3%)	
Former drinker	65 (5.2%)	563 (8.1%)		39 (2.1%)	280 (1.8%)	
Current drinker	978 (78.2%)	4963 (71.2%)		573 (30.1%)	5183 (32.9%)	
Physical activity (n, %)			0.0314			0.0528
Inactive	517 (41.4%)	2660 (38.1%)		899 (47.2%)	7073 (44.9%)	
Active	733 (58.6%)	4314 (61.9%)		1004 (52.8%)	8679 (55.1%)	
Menopausal status (n, %)						<0.0001
Premenopausal				498 (26.2%)	6137 (39.0%)	
Postmenopausal				1405 (73.8%)	9615 (61.0%)	

Continuous variables are presented as the mean ± standard deviation. Categorical variables are presented as frequencies (%). The *p*-values were based on Student’s *t*-test for continuous variables and the chi-square test for categorical variables. MetS, metabolic syndrome; PRS, polygenic risk score; BMI, body mass index; HDL, high-density lipoprotein.

**Table 3:** Demographic characteristics of study participants based on polygenic risk score (PRS) tertiles.

Variables	Men (*n* = 8224)			*p*-Value	Women (*n* = 17,655)			*p*-Value
	Low PRS (*n* = 2824)	Middle PRS (*n* = 2790)	High PRS (*n* = 2610)		Low PRS (*n* = 6018)	Middle PRS (*n* = 5831)	High PRS (*n* = 5806)	
Age (years)	54.7 ± 8.5	55.1 ± 8.4	54.8 ± 8.6	0.2308	52.5 ± 7.6	52.4 ± 7.6	52.3 ± 7.6	0.3253
BMI (kg/m^2^)	23.8 ± 2.5	23.8 ± 2.4	23.7 ± 2.4	0.3892	23.1 ± 2.6	23.1 ± 2.6	23.1 ± 2.6	0.5946
Waist circumference (cm)	83.4 ± 6.8	83.5 ± 6.6	83.3 ± 6.7	0.6324	76.4 ± 7.3	76.3 ± 7.3	76.2 ± 7.3	0.4637
Blood pressure								
Systolic blood pressure (mmHg)	123.4 ± 13.4	123.2 ± 13.0	122.9 ± 13.4	0.3561	119.0 ± 13.9	118.8 ± 14.2	118.6 ± 13.6	0.3993
Diastolic blood pressure (mmHg)	77.0 ± 9.2	76.5 ± 9.0	76.6 ± 9.1	0.0588	73.1 ± 9.2	73.0 ± 9.2	73.1 ± 8.9	0.6650
Triglyceride level (mg/dL)	116.8 ± 68.3	119.3 ± 70.8	131.4 ± 82.2	<0.0001	93.4 ± 45.1	96.9 ± 51.8	104.3 ± 58.9	<0.0001
Glucose level (mg/dL)	94.8 ± 18.6	94.5 ± 16.4	94.9 ± 18.0	0.6859	89.6 ± 12.1	90.0 ± 12.6	90.3 ± 12.9	0.0292
HDL cholesterol level (mg/dL)	52.4 ± 11.4	52.3 ± 11.8	51.2 ± 11.5	0.0002	59.0 ± 12.5	58.8 ± 12.6	57.8 ± 12.7	<0.0001
Education (n, %)				0.7673				0.6950
≤Elementary school	227 (8.0%)	215 (7.7%)	231 (8.9%)		917 (15.2%)	843 (14.5%)	855 (14.7%)	
Middle school	341 (12.1%)	351 (12.6%)	329 (12.6%)		1027 (17.1%)	995 (17.1%)	1031 (17.8%)	
High school	1155 (40.9%)	1125 (40.3%)	1053 (40.3%)		2765 (46.0%)	2685 (46.1%)	2618 (45.1%)	
≥College	1101 (39.0%)	1099 (39.4%)	997 (38.2%)		1309 (21.8%)	1308 (22.4%)	1302 (22.4%)	
Income (n, %)				0.0105				0.4873
Lowest	196 (6.9%)	210 (7.5%)	216 (8.3%)		538 (8.9%)	535 (9.2%)	545 (9.4%)	
Middle	1267 (44.9%)	1234 (44.2%)	1058 (40.5%)		2736 (45.5%)	2574 (44.1%)	2552 (44.0%)	
Highest	1361 (48.2%)	1346 (48.2%)	1336 (51.2%)		2744 (45.6%)	2722 (46.7%)	2709 (46.7%)	
Smoking status (n, %)				0.6076				0.2833
Non-smoker	809 (28.7%)	817 (29.3%)	713 (27.3%)		5823 (96.8%)	5678 (97.4%)	5634 (97.0%)	
Former smoker	1257 (44.5%)	1227 (44.0%)	1187 (45.5%)		87 (1.5%)	74 (1.3%)	73 (1.3%)	
Current smoker	758 (26.8%)	746 (26.7%)	710 (27.2%)		108 (1.8%)	79 (1.4%)	99 (1.7%)	
Alcohol intake (n, %)				0.3339				0.0547
Non-drinker	587 (20.8%)	575 (20.6%)	493 (18.9%)		3953 (65.7%)	3863 (66.3%)	3764 (64.8%)	
Former drinker	209 (7.4%)	206 (7.4%)	213 (8.2%)		105 (1.7%)	123 (2.1%)	91 (1.6%)	
Current drinker	2028 (71.8%)	2009 (72.0%)	1904 (73.0%)		1960 (32.6%)	1845 (31.6%)	1951 (33.6%)	
Physical activity (n, %)				0.7238				0.2510
Inactive	1093 (38.7%)	1091 (39.1%)	993 (38.1%)		2751 (45.7%)	2582 (44.3%)	2639 (45.5%)	
Active	1731 (61.3%)	1699 (60.9%)	1617 (62.0%)		3267 (54.3%)	3249 (55.7%)	3167 (54.6%)	
Menopausal status (n, %)								0.5502
Premenopausal					2246 (37.3%)	2174 (37.3%)	2215 (38.2%)	
Postmenopausal					3772 (62.7%)	3657 (62.7%)	3591 (61.9%)	

Continuous variables are represented as the mean ± standard deviation. Categorical variables are presented as frequencies (%). The *p*-values were based on the general linear model for continuous variables and the chi-square test for categorical variables. PRS, polygenic risk score; BMI, body mass index; HDL, high-density lipoprotein.

**Table 4:** Adjusted hazard ratios (HRs) and 95% confidence intervals (CIs) of metabolic syndrome (MetS) incidence based on oxidative balance score (OBS).

	OBS			
Men (*n* = 8224)	Person-years	Cases/Total	HR (95% CI)	*p*-Value
Quintile 1 (3.00–9.00)	6771.9	286/1633	1.00 (Ref)	
Quintile 2 (10.00–13.00)	6628.4	221/1623	0.90 (0.75–1.07)	0.2401
Quintile 3 (14.00–17.00)	6558.2	275/1591	1.05 (0.88–1.25)	0.6048
Quintile 4 (18.00–21.00)	6736.5	253/1626	0.96 (0.79–1.17)	0.6953
Quintile 5 (22.00–30.00)	7356.9	215/1751	0.83 (0.66–1.04)	0.1117
	OBS			
Women (*n* = 17,655)	Person-years	Cases/Total	HR (95% CI)	*p*-Value
Quintile 1 (4.00–10.00)	11,130.4	376/2730	1.00 (Ref)	
Quintile 2 (11.00–14.00)	14,410.6	361/3593	0.83 (0.72–0.96)	0.0136
Quintile 3 (15.00–19.00)	17,216.8	449/4218	0.83 (0.71–0.96)	0.0114
Quintile 4 (20.00–23.00)	14,761.0	374/3538	0.74 (0.63–0.88)	0.0005
Quintile 5 (24.00–30.00)	14,887.6	343/3576	0.74 (0.61–0.90)	0.0020

Adjusted for age, education, income, total energy, marital status, menopausal status, and regular exercise. MetS, metabolic syndrome; OBS, oxidative balance score; HR, hazard ratio; CI, confidence interval.

**Table 5:** Adjusted HRs and 95% CIs of metabolic syndrome incidence based on polygenic risk score (PRS).

	PRS			
Men (*n* = 8224)	Person-years	Cases/Total	HR (95% CI)	*p*-Value
Low PRS (0.00–5.32)	11,665.7	389/2824	1.00 (Ref)	
Middle PRS (5.32–7.24)	11,574.7	420/2790	1.10 (0.95–1.26)	0.1994
High PRS (7.24–17.77)	10,811.5	441/2610	1.27 (1.10–1.45)	0.0007
	PRS			
Women (*n* = 17,655)	Person-years	Cases/Total	HR (95% CI)	*p*-Value
Low PRS (0.82–5.37)	24,639.4	583/6018	1.00 (Ref)	
Middle PRS (5.37–7.30)	23,970.5	630/5831	1.15 (1.03–1.29)	0.0144
High PRS (7.30–17.69)	23,796.5	690/5806	1.28 (1.15–1.43)	<0.0001

Adjusted for age, education, income, total energy, BMI, marital status, menopausal status, and regular exercise. PRS, polygenic risk score; HR, hazard ratio; CI, confidence interval.

**Table 6:** Adjusted HRs and 95% CIs of metabolic syndrome (MetS) incidence based on weighted polygenic risk score (PRS) and oxidative balance score (OBS).

	Men (*n* = 8224)							
OBS	Low PRS (0.00–5.32)		Middle PRS (5.32–7.24)		High PRS (7.24–17.77)		*p* for trend	*p* interaction
	HR (95% CI)	*p*-Value	HR (95% CI)	*p*-Value	HR (95% CI)	*p*-Value		
Quintile 1 (3.00–9.00)	1.36 (0.96–1.91)	0.0820	1.28 (0.91–1.82)	0.1578	1.50 (1.07–2.11)	0.0193	0.5590	0.5124
Quintile 2 (10.00–13.00)	1.14 (0.80–1.63)	0.4610	1.01 (0.71–1.45)	0.9459	1.60 (1.14–2.24)	0.0060	0.0447	
Quintile 3 (14.00–17.00)	1.32 (0.95–1.83)	0.1027	1.42 (1.02–1.97)	0.0366	1.60 (1.16–2.19)	0.0039	0.8171	
Quintile 4 (18.00–21.00)	1.05 (0.75–1.47)	0.7749	1.55 (1.14–2.11)	0.0048	1.38 (0.99–1.92)	0.0565	0.0752	
Quintile 5 (22.00–30.00)	1.00 (Ref)		1.14 (0.82–1.58)	0.4403	1.33 (0.96–1.85)	0.0884	0.8958	
*p* for trend	0.0326		0.2090		0.4540			
	Women (*n* = 17,655)							
OBS	Low PRS (0.82–5.37)		Middle PRS (5.37–7.30)		High PRS (7.30–17.69)		*p* for trend	*p* interaction
	HR (95% CI)	*p*-Value	HR (95% CI)	*p*-Value	HR (95% CI)	*p*-Value		
Quintile 1 (4.00–10.00)	1.60 (1.18–2.17)	0.0025	1.71 (1.26–2.33)	0.0006	1.89 (1.40–2.56)	<0.0001	0.2315	0.4318
Quintile 2 (11.00–14.00)	1.11 (0.82–1.51)	0.4835	1.58 (1.18–2.13)	0.0021	1.67 (1.25–2.24)	0.0006	0.0024	
Quintile 3 (15.00–19.00)	1.40 (1.06–1.85)	0.0162	1.35 (1.02–1.79)	0.0370	1.52 (1.16–2.01)	0.0027	0.4519	
Quintile 4 (20.00–23.00)	1.11 (0.84–1.48)	0.4617	1.32 (1.00–1.73)	0.0510	1.43 (1.08–1.90)	0.0120	0.0497	
Quintile 5 (24.00–30.00)	1.00 (Ref)		1.26 (0.95–1.68)	0.1031	1.51 (1.15–1.98)	0.0027	0.0016	
*p* for trend	0.0712		0.1869		0.0091			

Adjusted for age, educational level, income, total energy, BMI, marital status, menopausal status, and regular exercise. *p* for trend values for linear trends were calculated by treating median value of each quintile or each PRS group as continuous variable. PRS, polygenic risk score; OBS, oxidative balance score; HR, hazard ratio; CI, confidence interval.

## Data Availability

The Korean Genome and Epidemiology Study (KoGES) data are available through a procedure described at https://biobank.nih.go.kr/cmm/main/mainPage.do (accessed 11 November 2024).
